# COVID-19 and Fake News in the Dominican Republic

**DOI:** 10.4269/ajtmh.20-0234

**Published:** 2020-04-29

**Authors:** Leandro Tapia

**Affiliations:** Instituto de Medicina Tropical & Salud Global, Universidad Iberoamericana, Santo Domingo, Dominican Republic

## Abstract

The first case of novel coronavirus disease (COVID-19) in the Dominican Republic coincided with a period of political crisis. Distrust in governmental institutions shaped the critical phase of early response. Having a weak public health infrastructure and a lack of public trust, the Ministry of Health (MoH) began the fight against COVID-19 with a losing streak. Within 45 days of the first reported case, the political crisis and turmoil caused by “fake news” are limiting the capacity and success of the MoH response to the pandemic.

The introduction of novel coronavirus disease (COVID-19) into the Dominican Republic was very untimely; the first case occurred during a chaotic period of political instability. After a failed election on February 15, 2020, distrust in public institutions rose due to a lack of clear explanation of the electoral crisis. This led to a month-long mass protest by thousands of people across the island-nation. The first confirmed case of COVID-19 occurred on February 29, 2020 in a traveler. During the next days, the Ministry of Health (MoH) announced the decision to enhance surveillance for COVID-19 and the designation of specific isolation centers across the country. Fifteen days after the first case was detected, the first diagnosis of autochthonous COVID-19 was confirmed. Six weeks after the first case was announced, the total burden of COVID-19 was 5,044 confirmed cases, with an estimated 4.8% mortality rate (based on 245 studied deaths).^1^

The Dominican Republic took measures to ensure early response to the COVID-19 crisis. When the WHO declared COVID-19 as a Public Health Emergency of International Concern,^2^ the Dominican Republic had no detected cases. When the WHO announced it as a pandemic, the Dominican Republic had only 11 detected cases, with about 50 possible cases identified by syndromic surveillance.^1,2^ Nighttime national lockdown measures were announced by the president just 17 days after the first detected case, at a time of 21 reported cases and one death,^1^ citing the experiences of previously affected areas.^3,4^ Citizens became wary of this measure, considering the extent of the economic impact of the lockdown and the low COVID-19 burden at the time. The general impression suggested an apparent overreaction by the authorities. This sentiment and government enforcement led to the detainment of thousands of civilians for breaking the curfew. Over time, social media increasingly denounced explanations by the MoH on why the epidemic curve had not yet flattened.

During the first week of the outbreak, the MoH gave morning press conferences to maintain official communication on the epidemic. These later evolved to not only report new cases and deaths but also provide recommendations for medical personnel and the general public.^1^ The MoH invested heavily on radio, social media, and television announcements to inform the population about the best preventive behaviors and symptom identification. Also, the MoH released a “National Protocol for the Diagnosis and Treatment for COVID-19”^5^ to ensure standardization of procedures for the diagnosis, care, and prevention of cases. However, all of these swift measures were not enough to regain the public’s trust and to stop the rapid spreading of “fake news” through the population.

The national protocol for the diagnosis and treatment of COVID-19 specifies that all prevention strategies focus on isolation techniques, use of personal protective equipment, and social distancing, and that treatments focus on symptomatic relief. Without local evidence for effective therapies, the general public and news outlets have looked internationally to seek experience with various treatments. Media outlets are circulating information on many studies of experimental treatments for COVID-19, including lopinavir/ritonavir,^6^ hydroxychloroquine,^7^ tocilizumab,^8^ and ivermectin,^9^ even though these studies are preliminary and show mixed results. Reports in the Dominican Republic have emerged showing that doctors are prescribing treatments and prophylaxis with regimens such as hydroxychloroquine plus azithromycin, tocilizumab,^10^ or ivermectin,^11^ all based on news reports rather than MoH direction. These prescription practices, without consequences for prescribers, demonstrate a lack of trust in public institutions and lack of regulation by the MoH. Up to mid-April, no clinical trial for COVID-19 treatment has been registered in the Dominican Republic, which could explain the use of experimental treatments, begging the question: Which Dominican institution regulates the actions of doctors that skew away from evidence-based guidelines?

Massive media bombardment regarding alternative COVID-19 treatments and increased numbers of doctors prescribing these treatments have led people to storm pharmacies and buy stocks of available drugs such as hydroxychloroquine.^10^ Thus, thousands of systemic lupus erythematosus (SLE) and rheumatoid arthritis (RA) patients are unable to access their treatment because of nationwide shortages.^12^ Social media posts from SLE and RA patients are frequently soliciting people to sell or donate hydroxychloroquine, which was purchased for COVID-19 prophylaxis.^13^ This state of affairs has highlighted two severe deficits of the Dominican Republic’s health system. First, sensationalist media can sway the opinions of medical personnel away from evidence-based practices. Second, with a culture of self-medication and lack of governmental regulation of drug use, drugs are purchased by clients without a prescription, and so without medical supervision.

Public speculation in the country, as in many parts of the world, has led to grocery shortages. Supermarkets and grocery stores have experienced scarcity of antibacterial gels, antibacterial wipes, detergents, and toilet paper.^14^ Rumors of the benefits of tonic water, for which quinine is an ingredient, led people to storm grocery stores in search of this product, which quickly became scarce.^15^ Shortages have not been limited to groceries. Pharmacies are experiencing shortages of essential items such as isopropyl alcohol, latex gloves, and medical-grade masks. Medical personnel have reported a lack of protective gear in hospital settings, including sites designated by the MoH as COVID-19 response sites, due to market shortages and inflation of costs, limiting hospital purchases.^16^ Overall, preventive actions against COVID-19 are a possibility only for those with adequate resources, not those at highest risk.

With the high stakes of the fight against the COVID-19 pandemic, what can a government without apparent regulatory capacity and public trust do to fight against it? What can medical practitioners in low-income settings do, when the system is rigged against their patients’ needs? What can medical practitioners do when the system cannot ensure their protection by providing necessary personal protective equipment? Answers to these questions are unclear, but what is clear is that the first step to regaining control of the response against the COVID-19 outbreak depends on the people’s engagement.

With increasing distrust in public institutions, health science academics should guide the COVID-19 narrative by identifying challenges faced by the people and acting as disinterested experts to solve them. Academics also need to publicly denounce wrongdoers and hold them accountable with scientific evidence. The job of a medical researcher has changed. Now, we need to communicate with the public and translate the current scientific literature into terms that can be understood and accessible, benefiting from the public’s increased interest. Furthermore, we need to become engaged in the solutions. Academics should take on the different social media platforms and attempt to silence those misinformed individuals helping to spread “fake news” by inspiring clinicians to ditch sensationalist media, and rather to search for answers within the scientific community. Medical personnel have to urge the authorities to scale-up preventive strategies, such as social distancing, and to uphold MoH regulations regarding drug use and misuse. Young researchers and emerging leaders have the opportunity to seize the moment and propose solutions. All of these suggestions seem obvious, but with a system that will not back up evidence-driven professionals, difficulties can arise. As an early-career medical doctor, I often wonder what happens when a patient comes to my office and leaves without understanding why the key to preventing a respiratory infection is social distancing and handwashing, when he has read something different online? What happens when a patient leaves hopeless, after I prescribed symptomatic treatment and home isolation, when he expected a specific treatment he heard about on the news? What happens when that patient finds his hopes satisfied by another doctor, who offers some unregulated experimental treatment? What happens when my reputation suffers because patients prefer an unproven treatment to evidence-based care? In reality, we cannot control the actions of every patient, but we have to fight on, for if we do not, the “fake news” will triumph over science.
